# sLORETA Source Localisation of Visual Mismatch Negativity in Dyslexic Children During Malay Orthographical Lexicon Stimulations

**DOI:** 10.21315/mjms2020.27.5.4

**Published:** 2020-10-27

**Authors:** Siti Atiyah Ali, Tahamina Begum, Faruque Reza, Nor Asyikin Fadzil, Faiz Mustafar

**Affiliations:** 1 Department of Neurosciences, School of Medical Sciences, Universiti Sains Malaysia, Kubang Kerian, Kelantan, Malaysia; 2 Department of Psychiatry, School of Medical Sciences, Universiti Sains Malaysia, Kubang Kerian, Kelantan, Malaysia

**Keywords:** event related potential, dyslexia, pseudoword, visual lexical processing, decision making

## Abstract

**Background:**

While there are studies on visual lexical processing in other languages among dyslexics, no studies were done in the Malay language. The origin of visual lexical processing might be different in the Malay language. We aimed to detect the source localisation of visual mismatch negativity (vMMN) during Malay orthographic lexicon stimulations, employing an event-related potential (ERP) study.

**Methods:**

Twelve dyslexic and twelve non-dyslexic children participated in this study. They pushed button ‘1’ when they saw real (meaningful) Malay words and button ‘2’ for pseudowords (meaningless). The source localisation of vMMN was performed in the grand average waveform by applying the standardised low-resolution brain electromagnetic tomography (sLORETA) method using Net Station software.

**Results:**

Left occipital (BA17) and left temporal (BA37) lobes were activated during real words in the non-dyslexic and dyslexic children, respectively. During pseudowords, BA18 and BA17 areas of the left occipital lobe were activated in the non-dyslexic and dyslexic children, separately. vMMN sources were found at the left temporal (BA37) and right frontal (BA11) lobes in non-dyslexic and dyslexic children, respectively.

**Conclusion:**

Right frontal lobe is the decision-making area where vMMN source was found in dyslexic children. We concluded that dyslexic children required the decision-making area to detect Malay real and pseudowords.

## Introduction

Reading is an ability that is mastered automatically by skilled readers, but in certain reading disabilities such as dyslexia, the readers exhibit poor mastery of reading skills, which can be due to reduced neural activation to word-like stimuli in certain regions of reading neural networks. Normal readers tend to read automatically without conscious effort in word recognition, particularly in discriminating words with different lexical structures (for example, between pseudowords and real words). Dyslexics have been said to have different visual lexical processing from that in non-dyslexics (1), especially in terms of discrimination and cognition towards lexical structures, hinting at the possibility of different sources of neural activity in dyslexics’ brains.

In a functional magnetic resonance imaging (fMRI) study in normal readers, it was reported that reading network activity was higher in the left inferior frontal gyrus of the frontal lobe, the left occipito-temporal region and the left temporo-parietal region during sight recognition processes while reading (2). According to Richlan (3), functional brain abnormalities in dyslexics were poorly activated in the left hemisphere-reading network in the occipito-temporal, inferior frontal and inferior frontal regions, which may be the cause for the poor automatic word recognition of the orthographic lexicon. However, the source activity of fast recognition for orthographical lexicons has yet to be determined. An event-related potential (ERP) is an electrophysiological technique that can provide objective spatial source localisation for automatical recognition and this is reflected in the mismatch negativity (MMN) component. MMN ERP component is different from other ERP components. MMN is defined as a negative waveform achieved by subtracting ERP responses from target to standard stimuli (4). The MMN is an automatic brain response to violations of regular/standard stimulation, and it can be evoked in auditory (5) and visual oddball tasks (6). Because MMN elicitation can be evoked by visual orthographic lexicon stimulations, it is worthwhile to know where the neural sources generate the automatic recognition of orthographical lexicons among dyslexics during the reading process, allowing the exploration of possible source abnormalities in dyslexics compared to that in normal readers. In this paper, visual MMN (vMMN) was referred to as the target source of MMN, as the MMN reflects stimulation elicited by the visual stimuli. Standardised low-resolution brain electromagnetic tomography (sLORETA) was chosen as the source localisation algorithm method for localising the source eliciting vMMN, as it can localise the strongest source (7) with less localisation errors (8).

Interestingly, different lexical structures of languages may elicit different vMMN processing, which has been observed in Chinese words (9) and in the Russian language (6), indicating that different structures of languages have different processing and neural source activities. Unfortunately, neither of these vMMN language studies focused on source localisation. Furthermore, to date, no studies have investigated the visual source localisation of automatic recognition using Malay orthographical strings as stimuli. Thus, this study aimed to investigate the source localisation of vMMN using the sLORETA method in response to the Malay orthographic lexicon in dyslexic school-aged Malay children.

## Study Design, Subjects and Screening Methods

### Study Design

This study is a quantitative cross-sectional and non-interventional study. A convenience sampling method was used for participants in both groups.

### Participants/Subjects

Twelve dyslexic and 12 healthy non-dyslexic primary school-aged children, with age ranging from 8–11 years, were recruited for the ERP study at the magnetoencephalography (MEG)/ERP Lab of Neurosciences in Universiti Sains Malaysia (USM). We calculated the sample size with Power and Sample Size (PS) software where the difference of means and standard deviations between non-dyslexic and dyslexic groups were 0.48 and 0.41, respectively. Therefore, the sample numbers were 12 in each group (non-dyslexic = 12 and dyslexic = 12) (10).

### Screening Methods

Three normal schools and three special schools, which were recognised by the Ministry of Education, Malaysia, were chosen to recruit the children for non-dyslexic and dyslexic groups, respectively. All schools were located in Kelantan, Malaysia. A Dyslexia Screening Instrument (DSI) was used to screen for dyslexia in both dyslexic and healthy children. DSI scaling revealed that all children in the non-dyslexic group received ‘0’ score and dyslexic children received score ‘1’. DSI was done by a school teacher (11). A psychiatrist excluded behavioural and cognitive issues like autism and attention deficit hyperactivity disorder (ADHD) in children of both groups with Mini-Mental State Examination (MMSE) child Malay version, developmental questionnaire form and ADHD assessment checklist.

All parents and participants provided their written informed consent before the experiments.

### Study Procedure and Stimuli Paradigm

Experimental stimuli were presented with E-Prime version 2.0 software (Psychology Software Tools, Inc., Sharpsburg, Pennsylvania, USA), and data acquisition was performed with Net Station software (Electrical Geodesics, Inc., Eugene, Oregon, USA). All participants sat in a dimly lit, sound-treated room, 80 cm away from a 22 in LCD computer where the stimuli were presented. A 128-electroencephalography (EEG) sensor net was used in the ERP study. Two to four-syllable words (real and pseudo Malay words) were presented for 500 ms with a 1000 ms interstimulus interval (ISI), in which the percentage of meaningful real words (standard stimuli) and pseudowords (target stimuli) was 80% and 20%, respectively. All participants pressed button ‘1’ for real meaningful words and ‘2’ for pseudowords.

### Procedure for sLORETA Source Analysis

vMMN source localisation was performed using Net Station software. First, the raw data were filtered from 0.3 Hz–50 Hz, with a 250-Hz stimuli rate. Filtered data were segmented from −100 ms–250 ms. All artefacts were removed using the artefact detection tool in Net Station software. Baseline correction was done at −100 ms, followed by the grand averages were performed for both groups. vMMN component (target stimuli – standard stimuli) was obtained after subtracting grand average waveforms between target and standard stimuli for both groups (4). Therefore, we found two vMMNs, one for the non-dyslexic group and another for the dyslexic group. To detect further source localisation of Malay real word and pseudowords induced vMMN, we selected a point of 202 ms which is a procedure to get source localisation, at the highest amplitude of vMMN to get clear source activation area within time range 150 ms–250 ms (4). After selecting the time point, the further process was done in the GeoSource tool of Net Station software.

**Figure 1:**
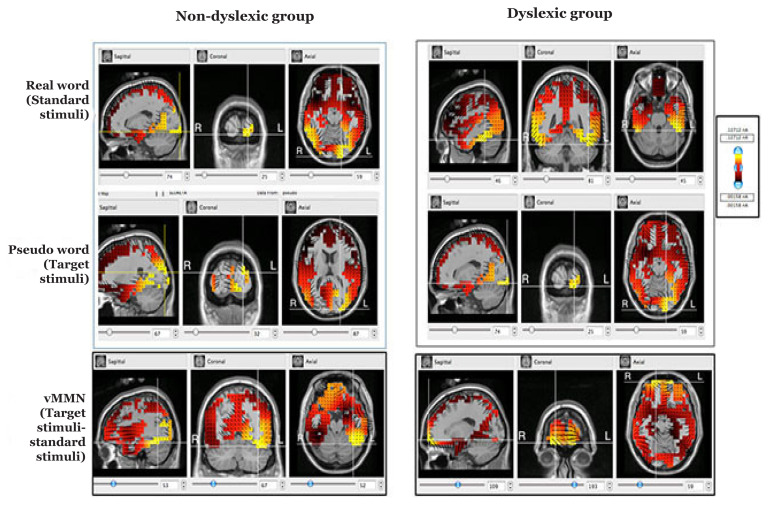
MRI images shown in sagittal, coronal and axial views for real Malay words (upper view), pseudowords (middle view) and vMMN (lower view) source localisation in non-dyslexic and dyslexic groups. Higher intensities of yellow colour indicate the active source location

In Net station software, we selected sLORETA method in the GeoSource tool for further source localisation. Computations of sLORETA were detected by using Montreal Neurological Institute (MNI)-152 template which can describe the anatomical locations in Brodmann areas (BA) (12). Therefore, after choosing MNI-152 followed by an ‘MRI image view’, the software automatically co-registers the data in an MRI image. Source localisation of vMMN was detected in three views of sagittal, coronal and axial in MRI images. Source activation areas of vMMN were detected with maximum intensity as yellow colour which can be compared between groups during real and pseudowords stimuli (Figure 1).

## Results

Sources of vMMN between non-dyslexic and dyslexic groups are shown in intensities, Brodmann areas, gyri, and brain lobes during the real and pseudoword stimuli conditions (Table 1). Figure 1 shows the sagittal, coronal and axial views of MRI under different stimuli between the groups. Each group shows three different sources for standard stimuli (real Malay words), target stimuli (pseudowords) and vMMN (the difference between real and pseudo words) (Figure 1, Table 1).

During real word stimulation, non-dyslexic group evoked source activation at BA17 and dyslexic group activated at BA37 which were in the left occipital lobe and left temporal lobe, respectively. However, during pseudoword stimulation both groups activated in nearly the same areas: non-dyslexic group activated in BA18 and dyslexic group evoked at BA17 and both Brodmann areas are located in the left occipital lobe (Table 1, Figure 1).

In this study, we aimed to explore vMMN source localisation in non-dyslexic and dyslexic groups. We found that the non-dyslexic group evoked a source of vMMN at BA37 (fusiform gyrus in the left temporal lobe) and the non-dyslexic group showed the source activation of vMMN at BA11 (superior frontal gyrus in the right frontal lobe) (Table 1, Figure 1).

## Discussion

This study aimed to investigate the sLORETA source localisation of vMMN using real and pseudo Malay words as stimuli in an ERP study between non-dyslexic and dyslexic children. We found that the source of vMMN was located in the right frontal lobe (BA11) in dyslexic children, which was different from those in non-dyslexic children (left temporal lobe: BA37).

Non-dyslexic children had BA17 activated during real-world stimuli, which is in the left occipital lobe (Table 1, Figure 1). This indicated that their visual attention for Malay real words was localised in the BA17 area (13). However, to read or detect real words, dyslexic children applied their spatial attention using BA37, which is an area of familiar word recognition (14). We assume that the activation area was diverted in dyslexic children during the Malay real words stimuli due to different brain connectivity.

We used Malay pseudowords as target stimuli in the present study. The detection of visual target stimuli requires more attention in the visual oddball paradigm. Non-dyslexic children have activated BA18, which is known as the visual association cortex (visual area 2) for word encoding (13). Considering these points, we can say that non-dyslexic children use their BA18 to understand and encode the Malay pseudoword. Based on the results shown in Table 1 and Figure 1, the dyslexic children in this study had BA17 (visual area1/primary visual cortex) activated when they implemented their visual attention for word encoding (13) when presented with pseudoword stimuli. Taking all of this information into consideration, we are certain that both non-dyslexic and dyslexic children required visual attention for word encoding during pseudoword recognition as they used visual association cortex/visual area 2 (BA18) and primary visual cortex/visual area1 (BA17), respectively (13).

However, our main goal of this study was to explore the vMMN source localisation which is the subtraction of pseudo and real words stimuli sources (target stimuli – standard stimuli). sLORETA source analysis revealed that non-dyslexic children activated BA37 which is fusiform gyrus in the left temporal lobe and dyslexic children activated BA11 which is superior frontal gyrus in the right frontal lobe during vMMN source localisation. BA37 is a spatial attention area and also an area of familiar word recognition (14) and BA11 is the decision-making area (15). We adopt the functions of these areas and assume that vMMN reflected through familiar word recognition areas for non-dyslexic children and decision-making areas for dyslexic children.

There are several ERP studies on different languages with real and pseudowords stimuli in dyslexic, normal healthy persons and different schizophrenic patients. But no study revealed source localisation of vMMN using sLORETA. Scalp sources were discovered using EEG electrodes in Russian real and pseudowords in healthy control participants and they found the vMMN source was in fronto-central areas for both real and pseudowords in the Russian language (6) (Table 1). Another Chinese study revealed that vMMN source results using Chinese real and pseudowords between native and non-native Chinese speakers. They found that native people evoked centro-parietal areas for real word and left temporal area for pseudowords. In contrast, non-native people activated the left parietal area for real words but no area was activated for pseudowords (9) (Table 1). However, both of these studies (6, 9) discovered their scalp source of vMMN using EEG electrodes which might be different from using the sLORETA procedure of our study as the techniques of source localisation are not same. There is a sLORETA source localisation of N170 but not vMMN in children with dyslexia (1). We need further study to explore the clear results and to compare vMMN source localisation among dyslexic children in different languages using word and pseudowords.

## Conclusion

We studied sLORETA source localisation of vMMN in dyslexic children using Malay real and pseudoword stimuli in an ERP study. Non-dyslexic and dyslexic children evoked BA37 and BA11 which were familiar word recognition and decision-making areas, respectively. As vMMN reflected visual attention, therefore, it is clear that dyslexic children used their visual attention using the decision-making area and non-dyslexic healthy children used familiar word recognition area to recognise Malay real and pseudowords. The results concluded that non-dyslexic children wanted to recognise Malay word and pseudowords as those are familiar or not but dyslexic children were making decisions about the words to understand about real or pseudowords.

## Figures and Tables

**Table 1:** sLORETA source localisations of vMMN were described with intensities, Brodmann areas, Gyri and lobes between non-dyslexic and dyslexic groups using Malay real words and pseudowords. Comparison of vMMN source locations of Malay real and pseudowords was shown with Russian and Chinese real and pseudowords

	sLORETA source localisation						EEG scalp source	EEG scalp source	

	Non-dyslexic group			Dyslexic group			Healthy group	Native	Non-native

	Malay real words	Malay pseudowords	vMMN (Malay words)	Malay real words	Malay pseudowords	vMMN (Malay words)	vMMN (Russian words) (6)	vMMN (Chinese words) (9)	
Intensities	0.152713 nA	0.167570 nA	0.026137 nA	0.080194 nA	0.089256 nA	0.049872 nA			
Brodmann areas	17 BA	18 BA	37 BA	37 BA	17 BA	11 BA			
Gyri	Lingual gyrus	Middle occipital gyrus	Fusiform gyrus	Inferior temporal gyrus	Lingual gyrus	Superior frontal gyrus			
Lobes	Left occipital lobe	Left occipital lobe	Left temporal lobe	Left temporal lobe	Left occipital lobe	Right frontal lobe	Real and pseudowords: Fronto-central area	Real word: Centro-parietal area Pseudowords: Left temporal area	Real words: Left parietal area Pseudowords: No effect
